# Iron Supplementation During Pregnancy- A Necessary or Toxic Supplement?

**DOI:** 10.1155/S156536330300013X

**Published:** 2003

**Authors:** Roberta J. Ward, Stephanie Wilmet, Rachida Legssyer, Robert R. Crichton

**Affiliations:** Unité de Biochimie Catholique Université de Louvain Louvain-la-Neuve 1348 Belgium

## Abstract

The effects of a single intramuscular iron dose, 10mg, to pregnant rats on Day of pregnancy, on the outcome of pregnancy, with respect to foetal weight and mother’s immune function has been investigated. Despite significantly elevated hepatic iron stores after iron supplementation in pregnant rats this had no significant effect upon blood haemoglobin or transferrin saturation levels. However the mean weight of the foetuses at Day 20-21 was significantly lower than that of the non-supplemented pregnant rats. Iron supplements significantly increased the activity of NADPH oxidase in the maternal alveolar macrophages, the primary event in the formation of the phagolysosome to combat invading organisms. However inducible nitric oxide synthase activity was significantly reduced in these macrophages as shown by decreases in LPSinduced and LPS+IFNγ-induced NOS activation. Iron supplementation to rats of normal iron status at the commencement of pregnancy did not show any beneficial effects to either the foetus or the mother.


### Iron Supplementation During Pregnancy-
### A Necessary or Toxic Supplement?

Roberta J Ward*, Stephanie Wilmet, Rachida Legssyer and Robert R. Crichton

*Unité de Biochimie, Catholique Université de Louvain*
*1348-Louvain-la-Neuve, Belgium*
*Phone: 0032 1047 2794; Fax : 0032 1047 2796*
*e-mail: ward@bioc.ucl.ac.be*

(Received December 2, 2002; Revision January 22, 2003; Accepted February 6, 2003)

### ABSTRACT

The effects of a single intramuscular iron dose, 10mg, to pregnant rats on Day 1 of pregnancy, on the outcome of pregnancy, with respect to foetal weight and mother's immune function has been investigated. Despite significantly elevated hepatic iron stores after iron supplementation in pregnant rats this had no significant effect upon blood haemoglobin or transferrin saturation levels. However the mean weight of the foetuses at Day 20-21 was significantly lower than that of the non-supplemented pregnant rats. Iron supplements significantly increased the activity of NADPH oxidase in the maternal alveolar macrophages, the primary event in the formation of the phagolysosome to combat invading organisms. However inducible nitric oxide synthase activity was significantly reduced in these macrophages as shown by decreases in LPS-induced and LPS+IFNγ-induced NOS activation. Iron supplementation to rats of normal iron status at the commencement of pregnancy did not show any beneficial effects to either the foetus or the mother.

### INTRODUCTION

Iron supplementation during pregnancy, particularly to mothers who are deemed to be iron deficient, has been proven to be of benefit to both mother and child; enhancing the iron stores in the former, to alleviate further iron losses that could occur during delivery or lactation while iron deficient anaemia is a risk factor for preterm delivery and low birth weight in the latter. However whether iron supplementation is necessary to

* Corresponding author.
Dr Roberta J Ward
Unité de Biochimie, Catholique Université de Louvain
1348 Louvain-la-Neuve, Belgium mothers who are deemed to have normal iron stores, i.e. ferritin >20μg/l haemoglobin >110g/l remains controversial. Since the additional iron requirement for a normal pregnancy is approximately 1g, (Table 1), [Letsky, 1991] it might be presumed that the lack of menstruation during the gestational period, (total iron gain =approximately 200μg) together with up-regulation of the proteins involved in iron absorption and transfer in the duodenal mucosa, i.e. DcytB, DMT1, Iregl and transferrin receptor, would be adequate to provide sufficient iron for the growth of the fœtus as well as maintaining the mother's iron stores. The routine use of iron prophylaxis during pregnancy is a subject of considerable debate, some advocating such treatment as routine [Horn, 1988] while others maintain that it should only be used when clinically indicated [Hibbard, 1988]. Iron supplementation of pregnant individuals with adequate iron status may aggravate oxidative stress [Lachili *et al.*, 2001], with the potential for oxidation of lipids and DNA [Schill and Reilly 2000], a factor which could contribute to preterm delivery, while the benefits could include the enhancement of the iron stores of the neonates [Haram, 2001], and be beneficial in its mental development.

In these present studies the effect of marginally increasing the maternal hepatic iron stores, approximately 2 fold, upon the outcome of pregnancy, i.e. birth weight and number of foetus, together with mother's iron stores and immune function has been studied in rats. Iron homeostasis was assessed by assay of hepatic iron stores in the mother at day 20-21, (total gestational time in rats =21-22 days), while the possible adverse effects of such a small increase in iron stores upon immune function was investigated in alveolar macrophage isolated from the mother at 21 days.

**Table 1**

Calculation of iron requirements for human pregnancy (Adapted from Letsky, 1991)

| | **Iron Requirement for Pregnancy (mg)** | **Range** |
|---|---|---|
| Foetus | 300 | (200-370) |
| Placenta | 50 | (35-100) |
| Expansion of maternal cell mass | 450 | (400-570) |
| Basal iron losses | 240 | (200-270) |
| **Total** | **1040** | **(835-1310)** |
| *Post delivery* | | |
| Contraction of maternal red cell mass | 450(gain) | (+400-570) |
| Maternal blood loss | 175 | (-100-250) |
| **Net iron cost of pregnancy** | **765** | |

If average = 44ml blood x 9 = 396 ml blood

13g haemoglobin in 100 blood

51.48g haemoglobin in 396 ml

1g haemoglobin contains 3.34mg Fe

51.48g haemoglobin contains **171.9 mg** iron

### EXPERIMENTAL

Female rats were mated overnight. Pregnancy was confirmed by the observation of sperm in the vaginal smear. A single intramuscular dose of iron, 10mg, in the form of iron dextran was administered to the rat on Day 1 of pregnancy. The pregnancy then proceeded for 14 or 21 days when the rat was anaesthetized with Nembutal, 0.5g/kg, and the alveolar macrophages removed by pulmonary lavage with 50 ml phosphate buffered saline. Blood was collected by cardiac puncture for haematological parameters. The livers were excised, the foetuses removed and weighed.

Alveolar macrophages were recovered after centrifugation at 1,200 rpm for 10 minutes, plated on plastic wells in Dulbecco's modified Eagle's medium (DMEM), containing 10% foetal calf serum (FCS), 100μg/ml streptomycin, 100U/ml penicillin, at 37°C under 5% $CO_2$. Cell viability was measured by trypan blue uptake extrusion (> 98%). Cells, 100,000/ml, were stimulated in medium containing lipolysaccharide, LPS, (1μg/ml) +/- IFNγ, 1U/ml for 44h. At the end of this time period the supernatant was removed for the spectrophoometric analysis of nitrite by Greiss reagent, the values being compared with nitrite standards in the range 1-50 μM.

In the remaining cells the activity of the enzyme NADPH oxidase was assessed by chemiluminescence using N-formylated methionylpeptide, FMLP, as the stimulant of its activity.

The liver iron content was assayed by inductively coupled plasma source after a small aliquot of liver, 100mg, had been homogenised with water, digested with concentrated nitric acid overnight, prior to a further addition of water.

The results are presented as mean ± standard deviations. Statistical analyses were by ANOVA with significance verified by Fisher 't' test.

### RESULTS

### Haematological results

Administration of iron had no effect upon the haematological results in the pregnant rats. Both the haemoglobin content, Figure 1a, and transferrin saturation, Figure 1b, showed similar and significant decreases in both groups whether iron supplemented or not. The transferrin saturation in the control group which had been supplemented with iron showed a significant increase.

### Outcome of pregnancy

#### *Foetus birth weight and numbers*

Table 2 shows that the mean foetal weight in the iron-supplemented mothers was significantly lower, $P<0.01$, than the non -supplemented rats. In addition, the number of placentas without a developing foetus increased approximately 2 fold in the iron supplemented rats, 7% by comparison to the non-supplemented, 3%. However the number of foetuses / dam was approximately 12 in both groups.

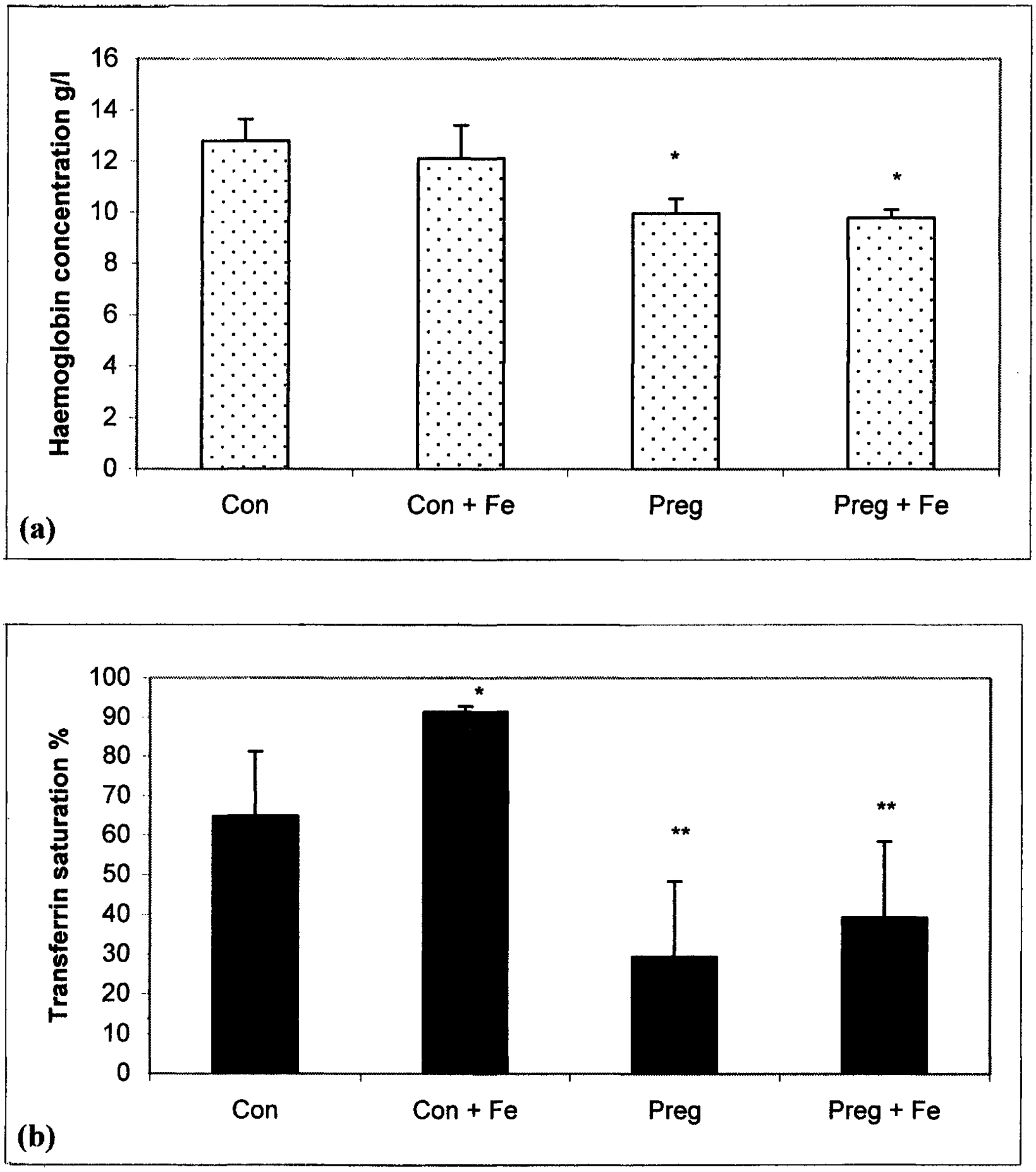

**Fig. 1:** Changes in haemoglobin , 1a, and transferrin saturation, 1b, after supplementation with iron, (10mg) or not on day 1 of pregnancy.

**Table 2.**

Foetus weights and numbers at day 20-21 of pregnancy +/- iron supplementation

| | G | No. of placenta | No. of foetus |
|---|---|---|---|
| Pregnant<br>n=12 | 4.502 + 0.88 | 138 | 134 |
| Pregnant + iron 10mg | 3.5772 + 1.37 | 60 | 56 |

### *Hepatic iron stores*

Table 3 shows the hepatic iron stores in the mothers supplemented with iron at 21 days of pregnancy. At 21 days the hepatic iron stores in the pregnant dams were significantly lower than the controls, by approximately 40%. In the supplemented pregnant group, the hepatic iron levels were considerably lower that the corresponding control group.

**Table 3**

Hepatic iron content at 14 and 21 days of pregnancy

| | µg/ g liver |
|---|---|
| *Controls* | 349 + 68 |
| Control + Iron 10mg | |
| 14 days | 536 + 71* |
| 21 days | 608 + 25* |
| *Pregnant* | |
| 14 days | 192 + 17** |
| 21 days | 212 + 18** |
| *Pregnant + Iron 10mg* | |
| 14 days | 595 + 20** |
| 21 days | 420 + 43** |

Statistical analyses by ANOVA with significance calculated between control and each of the treatments by Turkey 't' test.

### Immune function of maternal macrophages

### *(i) NADPH oxidase activity in alveolar macrophages*

Figure 2 shows the chemiluminescence activity in alveolar macrophages after stimulation with FMLP expressed as a percentage of the unstimulated control. Chemiluminescence was enhanced significantly in macrophages isolated from iron loaded and/ or pregnant rats after stimulation with FMLP by comparison to the unstimulated control macrophages.

### *(ii) NOS activity in alveolar macrophages*

Activation of control macrophages with LPS +/- IFNγ significantly increased the nitrite content of the culture media, Figure 3. Similar LPS +/- IFNγ - induced NOS activation was also assayed in the pregnant macrophages. However iron supplements significantly reduced NO release in the macrophages isolated from both the controls and pregnant rats, Figure 3.

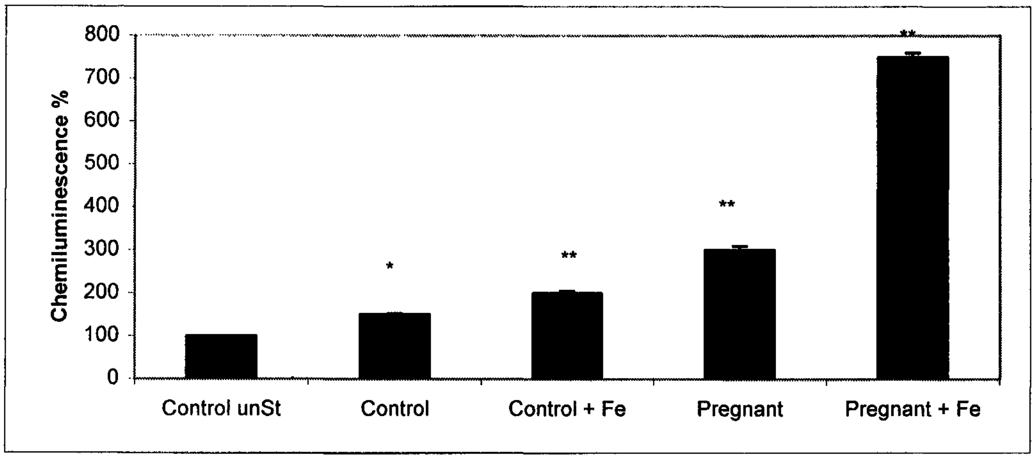

**Fig. 2:** Changes in chemiluminescence in alveolar macrophages isolated from rats which had been supplemented with iron (10mg )or not on Day 1 of pregnancy. Results are presented as % changes by comparison to unstimulated macrophages after stimulation with FMLP.

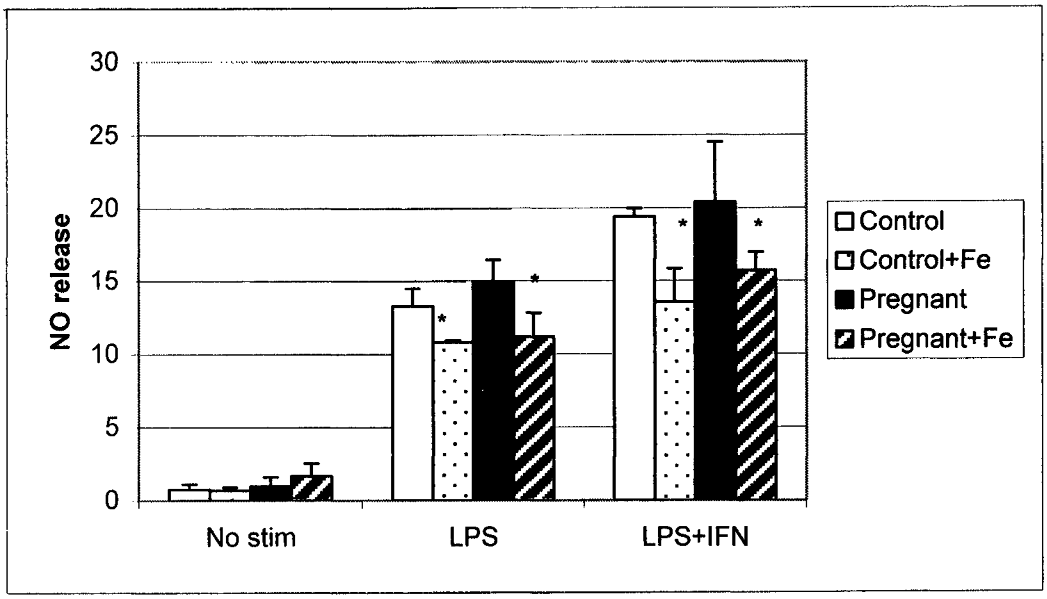

**Fig. 3:** Nitrite release from alveolar macrophages isolated from rats which had been supplemented with iron (10mg) or not on Day 1 of pregnancy after stimulation with lipopolysaccharide +/- interferon gamma

### DISCUSSION

The effect of iron supplementation upon the outcome of pregnancy in female rats with normal iron status at the commencement of pregnancy has been investigated in this present study. In the majority of previous investigations of animal models, primarily the rat, of the effects of iron supplements in pregnancy, the emphasis has been primarily on the effect of maternal iron depletion upon the developing foetus, e.g. low birth weights [Lewis *et al.*, 2001], and its consequence upon the offspring e.g. elevated blood pressure at 3 months [Lewis *et al.*, 2002].

The haematological results showed that iron supplementation at the commencement of pregnancy did not significantly alter parameters of iron adequacy, namely transferrin saturation and haemoglobin, despite the fact that the hepatic iron content in the supplemented pregnant rats was significantly higher than controls. However the literature states that these changes in blood parameters, caused by changes in blood volume with the resulting haemodilution as well as changes in red cell mass, are merely a normal physiological response to pregnancy and are not indicators of an underlying pathology.

The imposition of pregnancy in rats reduced the hepatic iron stores by approximately one third. Iron supplementation increased hepatic iron contents in the pregnant rats, which was lower than the corresponding controls indicating that iron had been utilised for the pregnancy. Human studies have identified an increased requirement for iron during pregnancy particularly during the 3$^{rd}$ trimesters of pregnancy [Blot *et al.*, 1999]. Macrophages play an important role both in storing excess iron and orchestrating the immune function; too much [Legssyer *et al.*, 2002] or too little [Hallquist *et al.*, 1992] will have an adverse effect upon their ability to respond to a toxic insult. In these present studies the effect of a marginal iron load on the ability of the macrophages to mount a defence, i.e. the activation of NADPH oxidase which is the first step in the formation of the phagolysosome, as well as their efficacy to combat invading organisms, i.e. activation of iNOS, was assessed. Interestingly macrophage NADPH oxidase activity was enhanced by pregnancy +/- iron in rats. This is in contrast to human studies where amelioration of inflammatory diseases occurs e.g. rheumatoid arthritis, which is attributed to lower NADPH oxidase activity [Crouch *et al.*, 1995]. Circulating female sex steroid hormones, oestrogens and progesterone, also play an important role in influencing their secretory profiles [Hunt *et al.*, 1998]. Progesterone acts as a powerful negative regulator of macrophages, reducing their ability to produce potent effector molecules such as nitric oxide, which could interfere with the success of pregnancy. [Hunt *et al.*, 1998]. Human polymorphonuclear leukocytes also showed lower NADPH oxidase activity during pregnancy, which could be due to a progressive change in circulating fatty acids, with a reduction in polyunsaturated FA, predominantly AA [Crocker *et al.* 2001], with significant reductions in respiratory burst compared with non pregnant controls; the responses to FMLP were reduced by 54% and to zymosan activated serum by 69% [Crouch *et al.*, 1995]. However in rats the induction of pregnancy had little effect upon LPS-induced or LPF-IFNγ-induced NO release, the results obtained being comparable to controls. However the iron supplement induced a significant decrease in NO release, which is compatible with our previous studies of marginal and chronic iron overload [Ward *et al.*, 2002].

Clearly these results indicate that the control of iron homeostasis is a crucial factor in pregnancy for both the mother and offspring. Iron supplements to rats which have normal iron status at the commencement of pregnancy appear to induce toxicity in both the mother – a reduced immune function – as well as to the offspring – reduced birth weight. In the literature, iron supplementation to women with normal iron status at the commencement of pregnancy remains a controversial issue. Whether it should be administered on a general or selective basis, or only during the third trimester when the need for additional iron is required [Blot *et al.*, 1999] is unclear. Further studies are underway in both animals and humans to clarify the advantages and disadvantages of iron supplements to individuals with normal iron status at the commencement of pregnancy upon both the mother and offspring.

### ACKNOWLEDGEMENTS

This work was supported by the European Union (QLK1-1999-00337) and the Fonds National de la Recherche Scientifique (Belgium), contract FRFC 2.4558.00.

### REFERENCES

1. E. Letsky, in *Clinical Physiology in Obstetrics* (F. Hytten and G. Chamberlain, Eds.), Blackwell Scientific Publications, 1991; 31.
2. E. Horn, *Br. Med. J.* **297**, 1325 (1988).
3. B.M. Hibbard, *Br. Med. J*,, **297**, 1324, 1988.
4. B. Lachili, I. Hininger, H. Faure, J. Arnaud, M.J. Richard, A. Favier and A.M. Roussel, *Biol. Trace Elem. Res*, **83**, 103 (2001).
5. T.O. Scoll and T. Reilly, *J. Nutr.* **130,** 443S (2000).
6. K. Haram, S.T. Nilsen and R.J. Ulvik, *Acta. Obstet. Gynecol. Scand.* **80**, 683 (2001).
7. R.M. Lewis, L.A; James J. Zhang, C.D. Bryne and C.D. Hales, *Br. J. Nutr.* **85,** 193 (2001).
8. R.M. Lewis, A.J. Forhead, C.J. Petry, S. Ozanne and C.N. Hales, *Br. J. Nutr.* **88**, 283 (2002).
9. I. Blot, D. Diallo and G. Tchernia, *Curr. Opin. Hematol*, **6,** 65 (1999).
10. R. Legysser, R. Ward, R.R. Crichton and J.R. Boelaert, *Biochem.Pharmacol*, **57**, 907, 1999.
11. N.A. Hallquist, L.K. McNeil, J.F. Lockwood and A.R. Sherman, *Am. J. Clin. Nutr* **55**, 741 (1992).
12. S.P. Crouch, I.P. Crocker and J. Fletcher, *J Immunol*, **155**, 5436 (1995).
13. J.S. Hunt, L. Miller and J.S. Platt, *Dev. Immunol*, **6**, 105 (1998).
14. I.P. Crocker, N. Lawson, P.N. Baker and J. Fletcher, *QJM*, **94** 475 (2001).
15. R.J.Ward, S. Wilmet, R. Legssyer and R.R. Crichton, *Biochem. Soc. Trans.* 762, 2002.

